# Chemokine CCL2 prevents opioid-induced inhibition of nociceptive synaptic transmission in spinal cord dorsal horn

**DOI:** 10.1186/s12974-021-02335-4

**Published:** 2021-12-02

**Authors:** Mario Heles, Petra Mrozkova, Dominika Sulcova, Pavel Adamek, Diana Spicarova, Jiri Palecek

**Affiliations:** Laboratory of Pain Research, Institute of Physiology, The Czech Academy of Sciences, Videnska 1083, 142 20 Praha 4, Czech Republic

**Keywords:** CCL2, TRPV1, MOR, Microglia, Synaptic transmission, Spinal cord, Hyperalgesia

## Abstract

**Background:**

Opioid analgesics remain widely used for pain treatment despite the related serious side effects. Some of those, such as opioid tolerance and opioid-induced hyperalgesia may be at least partially due to modulation of opioid receptors (OR) function at nociceptive synapses in the spinal cord dorsal horn. It was suggested that increased release of different chemokines under pathological conditions may play a role in this process. The goal of this study was to investigate the crosstalk between the µOR, transient receptor potential vanilloid 1 (TRPV1) receptor and C–C motif ligand 2 (CCL2) chemokine and the involvement of spinal microglia in the modulation of opioid analgesia.

**Methods:**

Patch-clamp recordings of miniature excitatory postsynaptic currents (mEPSCs) and dorsal root evoked currents (eEPSC) in spinal cord slices superficial dorsal horn neurons were used to evaluate the effect of µOR agonist [D-Ala^2^, N-Me-Phe^4^, Gly^5^-ol]-enkephalin (DAMGO), CCL2, TRPV1 antagonist SB366791 and minocycline. Paw withdrawal test to thermal stimuli was combined with intrathecal (i.t.) delivery of CCL2 and DAMGO to investigate the modulation in vivo.

**Results:**

Application of DAMGO induced a rapid decrease of mEPSC frequency and eEPSC amplitude, followed by a delayed increase of the eESPC amplitude, which was prevented by SB366791. Chemokine CCL2 treatment significantly diminished all the DAMGO-induced changes. Minocycline treatment prevented the CCL2 effects on the DAMGO-induced eEPSC depression, while mEPSC changes were unaffected. In behavioral experiments, i.t. injection of CCL2 completely blocked DAMGO-induced thermal hypoalgesia and intraperitoneal pre-treatment with minocycline prevented the CCL2 effect.

**Conclusions:**

Our results indicate that opioid-induced inhibition of the excitatory synaptic transmission could be severely attenuated by increased CCL2 levels most likely through a microglia activation-dependent mechanism. Delayed potentiation of neurotransmission after µOR activation is dependent on TRPV1 receptors activation. Targeting CCL2 and its receptors and TRPV1 receptors in combination with opioid therapy could significantly improve the analgesic properties of opioids, especially during pathological states.

## Background

Opioids remain the most effective analgesics for the treatment of many severe acute and chronic pain states. However, the administration of opioid analgesics often leads to serious side effects, including opioid tolerance, dependence, and hyperalgesia, limiting their clinical efficacy and therapeutic potential. Over the last decades, inhibitory mechanisms of opioids action on the first nociceptive synapse in the spinal cord dorsal horn were studied in great detail [1]. Much less clear are modulatory mechanisms that may counterbalance the opioid-induced inhibition after their application. Repeated opioid administration and consecutive opioid withdrawal often activate mechanisms characteristic for neuropathic pain and neuroinflammation. These are characterized by increased expression and release of chemokines, glial activation, and release of pro-nociceptive substances in the spinal cord that may counteract the analgesic effect [2, 3].

One of the prominent pro-nociceptive and inflammatory agents implicated in the modulation of neuropathic pain and opioid-induced analgesia is the C–C motif chemokine ligand 2 (CCL2) [4, 5]. CCL2 binds with the highest affinity to G protein-coupled C–C chemokine receptor type 2 (CCR2) and alters µ opioid receptor (µOR) function through a process of heterologous desensitization [6] similar to other chemokines [7]. The heterologous desensitization is mediated by phosphorylation of µOR, followed by rapid internalization of µOR. Exposure to CCL2 leads to a decrease of µOR function in dorsal root ganglia (DRG) neurons [8] and intrathecal (i.t.) administration of CCL2 neutralizing antibody augmented the opioid-induced analgesia [4].

Whether the effect of CCL2 is in fact direct, e.g., CCL2 acts directly on spinal neurons to reduce the µOR inhibitory effect, or acts indirectly through activation of microglia is not known. Microglia are considered important elements in the development of neuropathic pain and counter-regulators of opioid analgesia [9]. Several studies provided evidence of CCR2 in microglia, indirectly by observation of microglial activation after spinal administration of CCL2 [10], or directly by reporting immunoreactivity for the CCR2 in spinal cord microglia [11]. However, the development of a knock-in mouse with fluorescently labeled CCR2 led to a finding that monocyte-derived populations of cells expressing CCR2 within the central nervous system under naïve conditions can possibly be infiltrating monocytes, as they did not express CXCR1, a receptor expressed exclusively by microglia [12].

Another µOR internalization mechanism, the process of homologous desensitization, is initiated by phosphorylation of µOR induced by its agonist activation [13]. Activated µOR recruits β-arrestin 2 to the receptor and initiates internalization of the µOR via receptor phosphorylation by G protein-coupled receptor kinases (GRKs). Consequently, activation of µOR by morphine or DAMGO ([D-Ala^2^, N-Me-Phe^4^, Gly^5^-ol]-enkephalin) stimulates dissociation of β-arrestin 2 from transient receptor potential vanilloid 1 (TRPV1) and increases the sensitivity of TRPV1 [14, 15]. Ensuing cessation of opioid signaling leads to adenylyl cyclase activation and increase in cAMP, further stimulating TRPV1 sensitivity [16], confirmed for example by an increase in capsaicin-induced TRPV1 activity in dissociated DRG neurons [17].

In this study, the effect of CCL2 and TRPV1 antagonist SB366791 on µOR agonist DAMGO-induced inhibition was studied using behavioral tests and patch-clamp recordings of excitatory postsynaptic currents (EPSCs) in superficial dorsal horn neurons. The involvement of microglia activation in this process was evaluated by minocycline application [18]. A combination of the behavioral and electrophysiological data provides compelling evidence of opioids–chemokines–TRPV1 receptor crosstalk at the spinal cord level in opioid-induced analgesia and hyperalgesia.

## Methods

### Ethics statement

All animal care and experimental procedures were approved by the Institutional Animal Care and Use Committee and are consistent with the guidelines of the International Association for the Study of Pain and EU Directive 2010/63/EU for animal experiments.

### Spinal cord slice preparation

Male Wistar rats (P20–P23) were used for spinal cord slice preparation and subsequent electrophysiological recording (number of animals used for electrophysiology *n* = 53; mEPSC: CTRL *n* = 13, CCL2 *n* = 8, SB366791 *n* = 5, Minocycline + CCL2 *n* = 5; eEPSC: CTRL *n* = 7, CCL2 *n* = 5, SB366791 *n* = 5, Minocycline + CCL2 *n* = 5). Rats were anesthetized with isoflurane (3%), the lumbar spinal cord was removed and immersed in oxygenated ice-cold dissection solution containing (in mM) 95 NaCl, 1.8 KCl, 7 MgSO_4_, 0.5 CaCl_2_, 1.2 KH_2_PO_4_, 26 NaHCO_3_, 25 D-glucose and 50 sucrose. Animals were sacrificed by subsequent medulla interruption and exsanguination. Spinal cord segment was fixed to a vibratome stage (VT 1000S, Leica, Germany) using cyanoacrylate glue in a groove between two agar blocks. Acute transverse slices 300–350 μm thick were cut from L4 to L5 segments, incubated in the dissection solution for 30 min at 33 °C, stored in a recording solution at room temperature, and allowed to recover for 1 h before the electrophysiological experiments. Recording solution contained (in mM) 127 NaCl, 1.8 KCl, 1.2 KH_2_PO_4_, 2.4 CaCl_2_, 1.3 MgSO_4_, 26 NaHCO_3_, and 25 D-glucose. Slices used in the experiments with minocycline were incubated with minocycline alone for 15 min before the addition of CCL2. For the actual measurement, slices were transferred into a recording chamber that was perfused continuously with a recording solution (room temperature) at a rate of ~ 2 ml.min^−1^. All extracellular solutions were saturated with carbogen (95% O_2,_ 5% CO_2_) during the whole process.

### Patch-clamp recording

Patch-clamp recordings in spinal cord slices were made from superficial dorsal horn neurons in lamina I and II_(outer)_, similar to our previous experiments [19]. Individual neurons were visualized using a differential interference contrast (DIC) microscope (Leica, DM LFSA, Germany) equipped with a near-infrared-sensitive camera (Hitachi KP-200P, Japan) with a standard TV/video monitor. Patch pipettes were pulled from borosilicate glass tubing with resistances of 3.5–6.0 MΩ when filled with intracellular solution. The intracellular pipette solution contained (in mM): 125 gluconic acid lactone, 15 CsCl, 10 EGTA, 10 HEPES, 1 CaCl_2_, 2 MgATP, 0.5 NaGTP and was adjusted to pH 7.2 with CsOH. Voltage-clamp recordings in the whole-cell configuration were performed with an Axopatch 200B amplifier and Digidata 1440A digitizer (Molecular Devices, USA) at room temperature (~ 23 °C). Whole-cell recordings were low-pass filtered at 2 kHz and digitally sampled at 10 kHz. The series resistance of neurons was routinely compensated by 80% and was monitored during the whole experiment. AMPA receptor-mediated miniature and evoked EPSCs were recorded from neurons clamped at − 70 mV in the presence of 10 μM bicuculline and 5 μM strychnine. Miniature EPSCs were distinguished by the addition of 0.5 μM tetrodotoxin (TTX) to the bath solution. To record evoked EPSCs, a dorsal root was stimulated using a suction electrode with a glass pipette filled with an extracellular solution using a constant current isolated stimulator (Digitimer DS3, England). The intensity of the stimulation was adjusted to evoke stable EPSC with 0.5 ms stimulus duration and at least 3 × the minimal stimulus current at a frequency of 0.033 Hz.

## Materials

All chemicals used for extracellular and intracellular solutions were of analytical grade and purchased from Sigma Aldrich (St. Louis, MO, USA) and Tocris Bioscience (Bristol, UK). Capsaicin, SB366791 (Tocris Bioscience) were dissolved in dimethylsulfoxide (DMSO; Sigma Aldrich), which had a concentration of < 0.1% in the final solution. The concentration of SB366791 used for experiments was determined from IC_50_ = 7.5 ± 1.8 nM [20] and our earlier studies [21, 22]. The solution of CCL2 (R&Dsystems, Minneapolis, MN) was prepared using 0.1% BSA (Sigma-Aldrich) and the final concentration was defined according to the effective concentration used in our previous experiments [21]. DAMGO and minocycline (Sigma Aldrich) were dissolved in the recording solution to a final concentration based on previously published reports [23, 24].

### Data and statistical analysis

Software package pCLAMP 10 (Molecular Devices, USA) was used for data acquisition and subsequent offline analysis. Data segments of 2 min duration were analyzed for each experimental condition for the mEPSC recording. Only EPSCs with an amplitude of 5 pA or greater (which corresponded to at least twice the recording noise level) were included in the frequency analysis. The same events and data segments were used for amplitude analysis. The evoked EPSCs were recorded every 30 s, the averaged amplitude of 2 consecutive currents was used for further data analysis. Neurons with capsaicin-sensitive afferent input were identified by an increase of EPSC frequency (> 20%), measured after routine capsaicin (0.1 µM) application at the end of the experimental protocol.

Data were normalized as a percentage of the control value (100%) and expressed as means ± SEM. The control value for eEPSC was calculated from 4 control evoked currents before the DAMGO application. The control value for a group of neurons pre-treated with SB366791 was calculated from 4 evoked currents during SB366791 application before DAMGO application.

One Way ANOVA or One-Way repeated measures (RM) ANOVA followed by Bonferroni or Student–Newman–Keuls post hoc test were used for statistical comparisons of data with normal distribution and ANOVA non-parametric rank test or RM on ranks was used where appropriate. *P* value < 0.05 was considered statistically significant.

### Behavioral experiments

Adult male Wistar rats (250–300 g, *n* = 11) used in experiments were housed with a 12 h light/12 h dark cycle and in standard conditions with food and drinking water available ad libitum. The paw withdrawal latency (PWL) to thermal stimulation was tested using a plantar test apparatus (Ugo Basile, Italy) with radiant heat applied to the plantar surface of each hind paw. Rats were placed in nonbinding, clear plastic cages on a clear glass plate, with a heat source underneath. Rats were left to adapt to the testing environment for at least 15 min prior to any stimulation. Each paw was tested 4 times at each time point of the experiment with at least 5 min between the trials. Control values of PWL were acquired at the beginning of each experimental protocol before drug application. During the initial experiment a single DAMGO administration was given and the responses were evaluated. Two days later, the same animals received a single i.t. application of CCL2 followed by DAMGO 30 min later. PWL to thermal stimuli was assessed before CCL2 (control value) and after DAMGO application (at 30 min, 1, 2, and 4 h after injection). The averaged values from the hind paws of individual animals were averaged in the experimental groups.

For the i.t. delivery of 10 µl DAMGO (0.01 µg) or 10 µl CCL2 (1.4 µg) followed by 40 µl of saline, lumbosacral catheters were implanted between the L4 and L5 vertebrae a week before the experiment under isoflurane (3%) anesthesia [25]. The catheters were constructed from polyethylene tubing (PE5) and fixed by dental cement (Duracryl) to the vertebrae. The outer end of the catheter was fixed to the PE10 polyethylene tubing and externalized on the back of the animal. The position of the catheters was verified by a dye injection at the end of each experiment.

## Results

### DAMGO induced depression of mEPSC frequency was attenuated by CCL2

All EPSCs in this study were recorded from a population of nociceptive neurons predominantly localized in lamina I and II_(outer)_. The nociceptive input of the recorded neurons was verified by an increase of mEPSC or sEPSC frequency after application of capsaicin (0.1 µM) at the end of the experimental protocol.

**Fig. 1:**
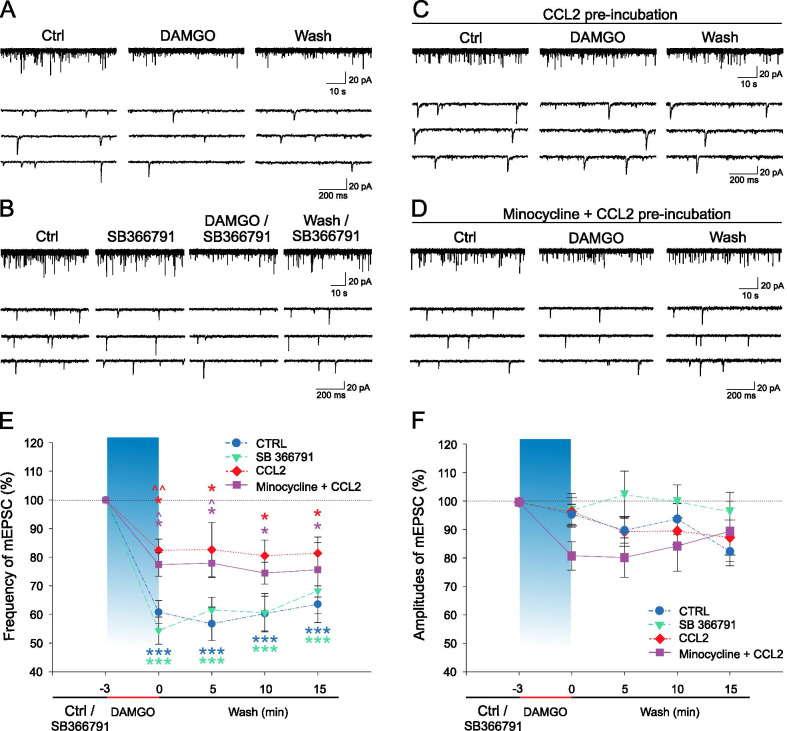
DAMGO induced decrease of mEPSC frequency was diminished after incubation with CCL2.** A**, **E** Application of DAMGO (1 µM, 3 min, *n* = 16) induced mEPSC frequency decrease that persisted during the wash-out period. **B**, **E** Pre-treatment with TRPV1 antagonist SB366791 (10 µM, 4 min, *n* = 8) did not significantly change the DAMGO-induced decrease of the mEPSC frequency. **C**, **E** Incubation of spinal cord slices with CCL2 (10 nM, 2 h) significantly attenuated DAMGO inhibitory effect (*n* = 11). **D**, **E** Inhibition of microglial activation by minocycline (100 µM) did not interfere with the CCL2 (10 nM, 2 h, *n* = 9) mediated attenuation of the DAMGO inhibition. **F** Mean mEPSC amplitudes were not significantly different between the experimental groups or after the DAMGO application. Statistical differences in each experimental group were identified using RM ANOVA on ranks followed by the Dunnett`s test; ^*^P < 0.05, ^***^P < 0.001 versus Ctrl/SB366791 part of the recording. Differences between the treatments were analyzed using one-way ANOVA followed by the Bonferroni test; ^*˄*^*P* < 0.05, ^*˄˄*^*P* < 0.01 versus CTRL group

During the control experiments, application of selective µOR agonist DAMGO (1 µM, 3 min) rapidly reduced mEPSC frequency to 60.8 ± 4.0% from the basal value (*P* ˂ 0.001, *n* = 16, Fig. 1A, E). This inhibition was most pronounced during the wash-out period 5 min after the cessation of DAMGO application (56.7 ± 5.9%, *P*˂0.001) and remained significant until the end of the recording (63.6 ± 6.4%, *P*˂0.001). Only 1 of the recorded neurons showed an increase in mEPSC frequency in the washout period (566%) and was excluded from the analysis, since the neuron did not respond to capsaicin. Mean amplitude of the mEPSCs was 19.3 ± 1.7 pA during the control period and was unaffected by the DAMGO application (95.2 ± 3.7% of the control, Fig. 1F) and through the washout (5 min 91.2 ± 4.6%, 10 min 94.7 ± 5.8%,15 min 82.8 ± 5.4%).

To test the possible role of TRPV1 receptors in the DAMGO-induced depression of the mEPSC frequency a specific TRPV1 antagonist SB366791 was used. Application of SB366791 (10 µM) started 4 min before the DAMGO (1 µM, 3 min) application and lasted till the end of the experiment. SB366791 alone did not change the frequency of the mEPSCs (90.9 ± 7.4%, *n* = 8, Fig. 1B). The response to the DAMGO in the presence of SB366791 elicited a decrease of mEPSCs frequency similar to the control experiments without the SB366791 pre-treatment (54.4 ± 4.7%, *P*˂0.001, Fig. 1B, E) and remained pronounced till the end of the recording (68.2 ± 8.0%, *P*˂0.001). Amplitude of the mEPSCs (16.5 ± 1.5 pA) was unchanged during the whole recording (SB: 91.3 ± 4.5%, DAMGO: 97.2 ± 6.0%, wash 5 min 102.6 ± 8.4%, 10 min 100.1 ± 6.0%, 15 min 96.6 ± 6.9%).

The effect of CCL2 was studied in spinal cord slices pre-incubated with CCL2 (10 nM, 2 h). In this group, DAMGO application decreased the mEPSCs frequency only to 82.4 ± 3.9% (*P* < 0.05, *n* = 11, Fig. 1C, E) from the basal value, and this decrease remained approximately at the same level in the subsequent 15 min of the wash-out period. The amplitude of the mEPSCs (16.8 ± 1.2 pA) did not change during the recording (DAMGO: 96.7 ± 5.5%, wash 5 min 91.1 ± 5.6%, 10 min 90.9 ± 5.0%, 15 min 89.2 ± 6.6%, Fig. 1F). These results indicate that CCL2 pre-treatment robustly attenuated the inhibitory effect of the DAMGO application on the mEPSCs frequency in the superficial dorsal horn neurons.

To assess the contribution of microglia to the observed CCL2-induced effect, spinal cord slices were pre-incubated with CCL2 together with minocycline, a blocker of microglia activation, for 2 h before the recording. Minocycline (100 µM) added to the incubation chamber 15 min before the CCL2 (10 nM) did not change the effect of CCL2 on the DAMGO-induced inhibition. Acute application of DAMGO decreased the mEPSCs frequency to 77.4 ± 4.1% (*P* < 0.05, *n* = 9, Fig. 1D, E), this decrease persisted during the wash-out period similar to what was observed in the CCL2 treatment group. The amplitude of the mEPSCs did not change during the whole recording (DAMGO: 81.0 ± 5.0%, wash 5 min 80.5 ± 7.0%, 10 min 84.5 ± 8.9%, 15 min 89.7 ± 10.7, Fig. 1F). Capsaicin application at the end of each recording was used to confirm the presence of the nociceptive input on the recorded neuron and induced robust increase of the mEPSC frequency (2567 ± 727%, *n* = 44). Our results suggest that microglial activation is not an essential mechanism in the CCL2-mediated attenuation of the DAMGO-induced mEPSCs frequency decrease.

### DAMGO induced effects on eEPSC amplitude were modulated by TRPV1 inhibition and by CCL2 application via microglia activation

**Fig. 2:**
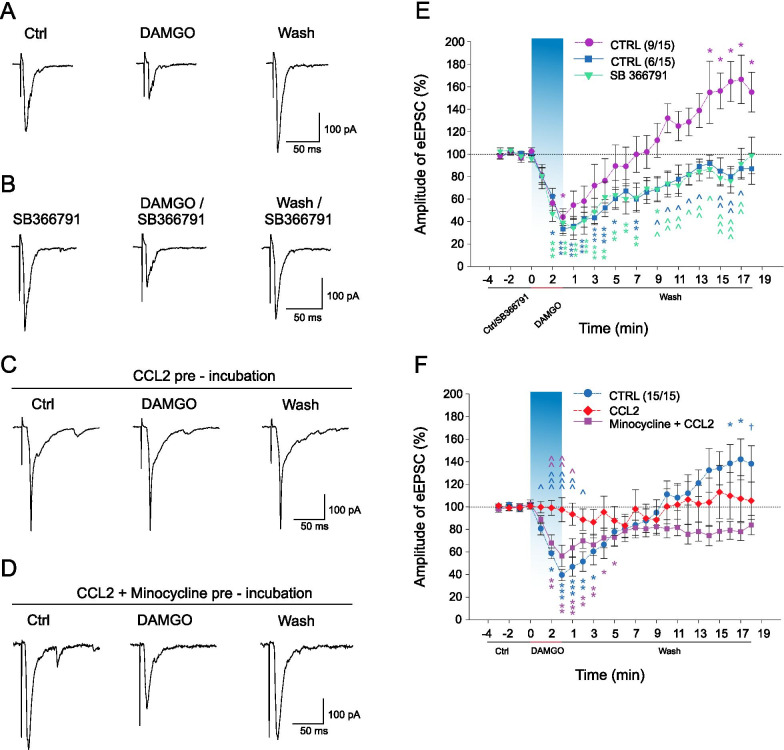
Effects of SB366791, CCL2, and minocycline on DAMGO induced depression and delayed potentiation of the eEPSC amplitude.** A**, **E** Application of DAMGO (1 µM, 3 min, *n* = 15) decreased the eEPSC amplitude in all of the recorded dorsal horn neurons. This depression was followed by an increase of eEPSC amplitude during the wash-out in 9 out of the 15 neurons. **B**, **E** Pre-treatment with TRPV1 antagonist SB366791 (10 µM, 4 min, n = 9) prevented DAMGO-induced increase of eEPSC amplitude during the washout. **C**, **F** Pre-incubation of spinal cord slices with CCL2 (10 nM, 2 h, *n* = 11) prevented both the DAMGO-induced decrease and the delayed increase of the eEPSC amplitude. **D**, **F** Inhibition of microglial activation with minocycline (100 µM, *n* = 8) prevented the blocking effect of CCL2 on DAMGO-induced decrease of the eEPSC amplitude. Statistical differences in each experimental group were identified using RM ANOVA followed by the Bonferroni test; ^*^P < 0.05, ^**^P < 0.01, ^***^P < 0.001 versus Ctrl/SB366791 part of the recording. Differences between treatments were analysed using one-way ANOVA followed by the Student–Newman–Keuls test; ^*˄*^*P* < 0.05, ^*˄˄*^*P* < 0.01, ^*˄˄˄*^*P* < 0.001 versus CTRL (E) or CCL2 group (**F**); ^+^*P* < 0.05 versus minocycline group

In the following experiments, dorsal root electrical stimulation was used to record evoked EPSCs that should reflect properties of synaptic transmission better than the mEPSCs. Altogether 15 neurons were recorded in the control group. All of them showed a significant amplitude decrease after the DAMGO (1 µM, 3 min) application, but differed by the amplitude changes during the washout period. In 9 of them, the DAMGO application induced a rapid decrease of the mean eEPSC amplitude (43.8 ± 7.4%, *n* = 9, *P*˂0.05, Fig. 2A, E) that returned to the basal value (99.8 ± 16.0%) in the 7^th^ min of the subsequent wash-out period. However, then the eEPSC amplitude continuously increased and reached 166.6 ± 21.5% (*P*˂0.01) in the 17th min of the wash-out period (Fig. 2E). The remaining 6 out of the 15 recorded neurons responded to the DAMGO application by a robust acute decrease of the eEPSC amplitude (33.0 ± 5.3%, *n* = 6, *P*˂0.001, Fig. 2E). This amplitude decrease was significantly different from the control value up to the 7^th^ min of the wash-out (59.9 ± 10.2%, *P*˂0.01) and then the eEPSCs returned close to the basal value.

Bath application of SB366791 (10 µM, 4 min) as a pre-treatment and together with the DAMGO application (1 µM, 3 min) was used to test the effect of TRPV1 receptors inhibition on the DAMGO-induced changes. Pre-treatment with SB366791 did not change the amplitude of the eEPSCs (90.0 ± 9.5%, *n* = 9). Subsequent DAMGO/SB366791 application again rapidly decreased the eEPSC amplitude (39.0 ± 9.8%, *P*˂0.001, Fig. 2B, E). Depression of the eEPSCs amplitude reached its maximum in the 1st min of the wash-out period (34.3 ± 10.2%, *P*˂0.001, Fig. 2E) and remained significantly different from the control value up to the 9th min of the wash-out (68.6 ± 10.3%, *P*˂0.05). Amplitude of the eEPSC continually increased and eventually reached the control value in the 18th min of the wash-out (98.8 ± 16.3%, Fig. 2E). However, the delayed potentiation of the eEPSC amplitude was not present in any of the recorded neurons. Thus inhibition of the TRPV1 receptors prevented the DAMGO-induced delayed increase of the eEPSCs amplitude.

The possible role of CCL2 was tested in spinal cord slices after pre-incubation with the CCL2 (10 nM, 2 h) in another group of neurons. Acute application of DAMGO (1 µM, 3 min) after the CCL2 pre-treatment did not induce decrease of the eEPSCs amplitude (97.4 ± 10.7%, *n* = 11, Fig. 2C, F). The amplitude did not change significantly throughout the whole recording. Hence the incubation with CCL2 prevented not only the acute DAMGO-induced depression but also the eEPSC amplitude potentiation present during the washout in the subpopulation of the neurons recorded under the control conditions.

Inhibition of the glial cells was tested similarly to the mEPSCs experiments. Spinal cord slices were pre-incubated with minocycline (100 µM) and CCL2 (10 nM, 2 h) simultaneously, while the minocycline was added 15 min before the CCL2. Acute application of DAMGO on neurons in these slices significantly decreased the eEPSC amplitude (56.4 ± 8.4%, n = 8, *P* < 0.001, Fig. 2D, F). In contrast to the DAMGO application in naive slices, the amplitudes of the eEPSCs during the wash-out period did not exceed the control value (Fig. 2D, F). Mean control eEPSC amplitudes (recorded before the DAMGO application) were not significantly different between the experimental groups (CTRL: − 433.9 ± 163.1 pA; SB366791: − 497.1 ± 123.6 pA; CCL2: − 392.3 ± 133.7 pA; Minocycline + CCL2: − 744.5 ± 206.4 pA). Our results show that minocycline treatment significantly attenuated the effect of CCL2 on the DAMGO-induced eEPSC amplitude depression, while the increase of the eEPSCs amplitude during the washout period was absent.

### CCL2 attenuates DAMGO-induced hyposensitivity to thermal stimuli in vivo

**Fig. 3:**
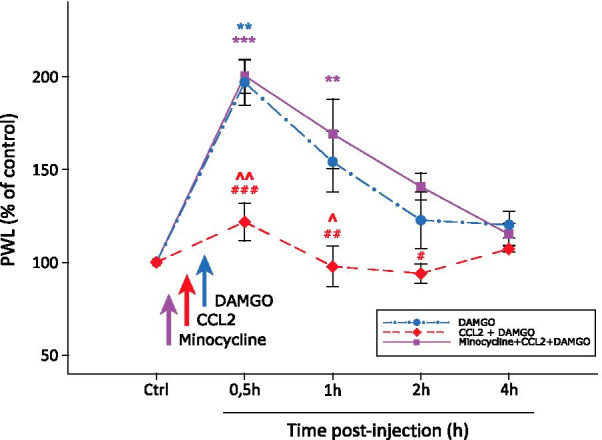
Thermal hyposensitivity induced by intrathecal application of DAMGO was attenuated by pre-treatment with CCL2 in a microglia activation-dependent manner. Acute i.t. application of DAMGO (0.01 µg, *n* = 5) increased the PWL to thermal stimuli. A single i.t. CCL2 (1.4 µg, *n* = 5) pre-treatment 30 min before the i.t. application of DAMGO (0.01 µg) prevented the DAMGO-induced increase of the PWL and the PWL remained unchanged throughout the testing period. Single i.p. application of minocycline (45 mg/kg, *n* = 6) prevented the CCL2-induced effect on the DAMGO-evoked thermal hypoalgesia in all of the tested animals. Statistical differences in each experimental group were identified using RM ANOVA on ranks followed by the Dunnett`s test; ^*^P < 0.05, **P < 0.01, ^***^P < 0.001 versus Ctrl. Differences between treatments were analyzed using one-way ANOVA followed by the Bonferroni test. ^*˄*^*P* < 0.05, ^*˄˄*^*P* < 0.01 versus DAMGO group; ^*#*^*P* < 0.05, ^*##*^*P* < 0.01, ^*###*^*P* < 0.001 versus Minocycline + CCL2 + DAMGO group

We used behavioral experiments to determine whether the in vitro observed effects of CCL2 on the DAMGO-induced inhibition of the excitatory synaptic transmission translates into modulation of DAMGO induced analgesia (Fig. 3). An intrathecal catheter was used for the DAMGO (0.01 µg) application with or without a single acute i.t. CCL2 (1.4 µg) pre-treatment. Acute DAMGO i.t. injection significantly prolonged PWL to thermal stimuli 30 min after the application (196.9 ± 12.5% of the control PWL, n = 5, *P* < 0.01; Fig. 3). Thermal hyposensitivity decreased 1 h after the DAMGO application (154.2 ± 16.2%) and diminished 2 h later (120.2 ± 7.3%). In further experiments, the i.t. application of CCL2 (1.4 µg) was used as a pre-treatment 30 min before the DAMGO i.t. (0.01 µg) application. In this case DAMGO application failed to significantly prolong PWL to the thermal stimuli in any of the assessed time points (30 min: 121.7 ± 10.2%, 1 h: 97.7 ± 11%, 2 h: 94 ± 5.2%, 4 h: 107.2 ± 1.7%, n = 5). Thus, pre-treatment with CCL2 was able to completely diminish the analgesic effect induced by spinal µOR activation.

To assess the possible influence of microglial activation on the CCL2 induced attenuation of the DAMGO-mediated analgesic effect, we applied a single i.p. dose of minocycline (45 mg/kg) 1 h before the CCL2 pre-treatment. Following DAMGO application significantly prolonged PWL to thermal stimuli in all of the tested animals (30 min: 200.5 ± 9.5%, *P* < 0.001; 1 h: 169.2 ± 7.6%, *P* < 0.01; 2 h: 140.8 ± 5.9%; 4 h: 115.1 ± 5.5%, *n* = 6). Minocycline thus prevented the effect of CCL2 on DAMGO induced analgesia.

## Discussion

Our results showed that activation of spinal µORs by DAMGO induced a significant inhibition of excitatory synaptic transmission and had an analgesic effect in behavioral experiments. In a subpopulation of the recorded neurons, the amplitude of the dorsal root stimulation evoked EPSCs increased following the cessation of the DAMGO application and this potentiation was dependent on TRPV1 receptors activation, suggesting their possible role in opioid-induced hyperalgesia. Pre-treatment with chemokine CCL2 blocked the DAMGO-induced in vitro inhibition, delayed potentiation and in vivo analgesia. CCL2 induced inhibition of DAMGO effects on the eEPSC amplitude and behavioral analgesia were prevented by minocycline treatment, suggesting a role of microglial activation.

### Effect of DAMGO on synaptic transmission and analgesia

Application of DAMGO during the patch-clamp recordings from neurons in spinal cord slices induced a significant decrease of the mESPC’s frequency and dorsal root eEPSC’s amplitude as was demonstrated previously [23, 26]. This inhibitory effect in Aδ- and C-fiber terminals is mediated mainly by presynaptic µORs and involves inhibition of N-type and P/Q type voltage-dependent Ca^2+^ channels and direct inhibition of neurotransmitter release machinery [26]. We have observed a robust increase of the eEPSC’s amplitudes in 60% of the neurons at the end of the washout period. This is in agreement with earlier observation in adult rats [23]. However, *Zhou *et al*.* also reported an increase of the mEPSC frequency following µOR activation in 50% of the recorded lamina II neurons. We have recorded a similar increase of mEPSC frequency only in 1 out of the 16 recorded superficial dorsal horn neurons. This difference may be due to different experimental conditions, such as the age of the animals, recording temperature and different populations of the recorded neurons.

µORs in the rat dorsal horn are distributed both post- and presynaptically [27, 28]. Major postsynaptic effectors of opioid-induced analgesia are G protein-coupled inwardly rectifying potassium channels (GIRKs) that contribute to the analgesic effect by a postsynaptic decrease in excitability [29]. However, several studies done in the rat dorsal horn suggest that OR agonists rely primarily on presynaptic mechanisms for their inhibitory effect on glutamatergic synaptic input to substantia gelatinosa neurons. Opioids in concentrations sufficient to attenuate synaptic transmission caused no significant change of holding currents in substantia gelatinosa neurons [30] and had no significant effect on inward glutamate-induced currents, indicated by unchanged amplitudes of mEPSCs [30]. In addition experiments on GIRK2-null mice showed that the absence of GIRK2 did not eliminate the analgesic effect of systemic morphine and higher doses produced maximal analgesic effect even in mutant animals [31]. Taken together, our findings are in agreement with other studies of opioid-induced inhibition in the dorsal horn and we assume that the DAMGO-induced depression of mEPSCs frequency was primarily a presynaptic event, based on the voltage-clamp configuration of the recorded neuron and no change in the mEPSCs amplitude. While the situation in eEPSC recordings and especially during the behavioral experiments was more complex and postsynaptic µOR could be partially responsible for the inhibitory effects of DAMGO we believe that presynaptic mechanisms were dominant.

The delayed potentiating effect of DAMGO on the eEPSC’s amplitude was prevented by TRPV1 antagonist application suggesting dependence on the TRPV1 activation. This finding corresponds with a prominent role of TRPV1 expressing primary afferents in opioid-induced long-term potentiation [23]. Various factors could contribute to the activation of TRPV1 receptors in our experimental conditions, such as endogenous agonists and changes in membrane potential [22]. The activation of TRPV1 following the DAMGO application could be enhanced by TRPV1 sensitization mediated by dissociation of beta-arrestin-2 from the receptor [14]. In our experiments, the dorsal root stimulation-induced action potential propagation and changes in the presynaptic membrane voltage could play a role in the TRPV1 activation. During the mEPSC recordings, the absence of membrane depolarization might decrease the probability of TRPV1 activation after the DAMGO application. Our results suggest that activation of presynaptic TRPV1 in the primary afferent fibers, terminating in the lamina I and II of the superficial dorsal horn, mediated the DAMGO-induced potentiation of eEPSCs.

The behavioral experiments supported the in vitro experiments and demonstrated a vigorous analgesic effect in the heat-induced paw withdrawal test after the DAMGO i.t. application. We did not observe any signs of opioid-induced hyperalgesia during the time course of the experiment that would reflect the potentiating effect of DAMGO application on the eEPSCs amplitude. Opioid-induced hyperalgesia usually occurs after high-dose opioid application or after abrupt opioid withdrawal after prolonged administration of fast-acting opioids [32]. Our behavioral observations suggest that a single dose of i.t. DAMGO application did not lead to opioid-induced hyperalgesia.

### Effect of CCL2 on opioid-induced synaptic depression and analgesia

Our study shows that spinal cord slices incubation with CCL2 prevented both acute opioid-induced depression and also subsequent delayed EPSC’s potentiation, plausibly due to µORs desensitization/internalization [8]. These findings were further supported by behavioral experiments, where i.t. pre-treatment with CCL2 diminished DAMGO-induced analgesia.

A steadily rising number of studies indicate a critical role of CCL2 and its receptor CCR2 in nociceptor sensitization and pain hypersensitivity after inflammation or nerve injury [33, 34]. Upregulation of CCL2 expression in the spinal cord and primary sensory neurons in DRG was shown in several peripheral nerve injury models [5, 35, 36] and spinal administration of CCL2 neutralizing antibody attenuated mechanical allodynia and heat hyperalgesia in mice after spinal nerve ligation [35]. Our previous work showed that CCL2 alone can increase spontaneous glutamate release from the central endings of nociceptive primary afferent fibers as well as amplitudes of evoked EPSC’s in a subpopulation of dorsal horn neurons and that spinal administration of CCL2 induced thermal hyperalgesia and mechanical allodynia [21].

CCL2 diminished the opioid-induced inhibition of nociceptive transmission, presumably by internalization of presynaptic µOR. The inhibitory effect of CCL2 on the µOR function in our experiments is supported by previous observation that exposure to several chemokines including CCL2 can significantly diminish the function of µOR in DRG cultured neurons [8]. Heterologous desensitization, a process essential for the function of any G protein-coupled receptor, was studied intensively between µOR and different chemokines especially in the immune system [7]. It was suggested that agonist activation of CCR2 leads to activation of downstream cascades that cross-desensitize µOR and may lead to opioid analgesia impairment. This is supported also by a study, where CCR2 antagonist enhanced opioid analgesic potency in neuropathic rats [37]. The effect of CCL2 on the DAMGO-induced inhibition seemed to be less pronounced on the mEPSC frequency compared to the effect on the eEPSC amplitude. It is likely that the DAMGO-induced depression of glutamate release and CCL2-induced µOR desensitization had a greater effect during the eEPSC recordings, where voltage-gated calcium channels are massively activated by the action potential.

### Role of microglia in the CCL2-induced modulation of µOR function

Minocycline blocked the effect of CCL2 on µOR function in both in vitro and in vivo experiments. In recordings of the eEPSC, pre-incubation with minocycline completely restored the inhibitory effect of DAMGO and minocycline pre-treatment prevented CCL2-induced µOR desensitization in behavioral experiments. These data suggest that the CCL2 inhibitory effect was dependent on activation of microglia—key players in modulation of nociceptive signaling in the spinal cord. Despite its implication in a number of pathogeneses, the cellular distribution of CCR2 remains controversial. Older studies in rats report CCR2 mRNA, protein, and effect of CCL2 on electrophysiological properties of both cultured neurons and neurons in acute spinal cord slices [21, 38]. A study that relied mostly on conventional immunohistochemistry showed that CCR2 is exclusively expressed by astrocytes [39]. The development of the CCR2-reporter mouse line allowed direct detection of CCR2 protein distribution [12]. Reports from this mouse strain show no detectable CCR2 in resident cells of the lumbar spinal cord in healthy mice, while CCR2 expression expanded from infiltrating monocytes to microglia and neurons with the progression of amyotrophic lateral sclerosis [40]. In the context of neuropathic pain, minocycline blocked microglial activation, inhibited CCL2-induced potentiation of glutamatergic transmission in substantia gelatinosa neurons, and attenuated hyperalgesia induced by spinal injection of CCL2 [41]. Despite the controversy, CCL2-induced activation of microglia played most likely a significant role in our experiments. This could be explained by the ability of CCL2 to elevate levels of other pro-nociceptive substances, such as IL-1β, IL-6, and IL-18 that can, in turn, lead to the activation of microglia [37]. Activated microglia might then amplify the process by the release of additional chemokines leading to µOR inhibition.

While minocycline prevented the CCL2-induced effect on µOR in eEPSC recordings and in vivo, it failed to counteract the DAMGO-induced inhibition of mEPSC frequency in the presence of CCL2. This suggests that CCL2 attenuates opioid-induced depression of spontaneous glutamate release (mEPSC) through direct action on presynaptic neurons, without the microglia involvement. However, during the action potential mediated glutamate release (eEPSC) and in vivo was this mechanism insufficient and glia activation played a role. Microglia upon activation secrete a large variety of substances including but not limited to various cytokines [42]. We assume that CCL2-induced release of other substances from microglia was necessary to prevent the effect of DAMGO during the eEPSC recordings and behavioral experiments.

## Conclusion

Here we report that CCL2 application prevented depression of excitatory synaptic transmission in the spinal cord induced by activation of µOR, thus dramatically reducing the opioid analgesic effect. This CCL2-mediated effect on opioid-induced analgesia was largely dependent on microglial activation. The inhibition of the excitatory synaptic transmission at the first synapse in the pain pathway induced by activation of spinal µORs also induced a delayed increase of eEPSC’s in some neurons that was dependent on presynaptic TRPV1 receptors activation. Our results suggest that opioid-mediated analgesia may be attenuated in pathological states, where increased levels of CCL2 are present, such as neuropathic pain conditions. We suggest that targeting the crosstalk between the OR, CCR2, TRPV1 receptors and inhibition of microglial activation could significantly improve the analgesic properties of opioids.

## Data Availability

All data supporting the conclusions of this study are presented in the manuscript. The data sets analyzed for the current study are available from the corresponding author upon a reasonable request.

## Abbreviations

µOR: µ Opioid receptor
TRPV1: Transient receptor potential cation channel subfamily V member 1
EPSC: Excitatory postsynaptic currents
DAMGO: [D-Ala2, N-Me-Phe4, Gly5-ol]-enkephalin
CCL2: C–C motif chemokine ligand 2
CCR2: C–C chemokine receptor type 2
DRG: Dorsal root ganglia
i.t.: Intrathecal
GRK: G protein-coupled receptor kinase
TTX: Tetrodotoxin
RM ANOVA: Repeated measures analysis of variance
PWL: Paw withdrawal latency
i.p.: Intraperitoneal
